# Integrated analysis of single-cell, spatial and bulk RNA-sequencing identifies a cell-death signature for predicting the outcomes of head and neck cancer

**DOI:** 10.3389/fimmu.2024.1487966

**Published:** 2024-11-07

**Authors:** Yue Pan, Lei Fei, Shihua Wang, Hua Chen, Changqing Jiang, Hong Li, Changsong Wang, Yao Yang, Qinggao Zhang, Yongwen Chen

**Affiliations:** ^1^ Institute of Immunology, People’s Liberation Army (PLA), Third Military Medical University, Chongqing, China; ^2^ Department of Pharmacy, The General Hospital of Western Theater Command, Chengdu, Sichuan, China; ^3^ Chongqing Renpin Otolaryngology Head and Neck Surgery Hospital, Chongqing, China; ^4^ Department of Pathology, People’s Liberation Army Joint Logistic Support Force 989^th^ Hospital, Luoyang, Henan, China; ^5^ Chronic Disease Research Center, Medical College, Dalian University, Dalian, Liaoning, China

**Keywords:** HNC, cell death, machine learning, risk score, spatial transcriptomics

## Abstract

**Background:**

Cell death plays an essential role in carcinogenesis, but its function in the recurrence and postoperative prognosis of head and neck cancer (HNC), which ranks as the 7^th^ most common malignancy globally, remains unclear.

**Methods:**

Data from five main subtypes of HNC related single-cell RNA sequencing (scRNA-seq) were recruited to establish a single-cell atlas, and the distribution of cell death models (CDMs) across different tissues as well as cell subtypes were analyzed. Bulk RNA-seq from the Cancer Genome Atlas Program (TCGA) dataset was subjected to a machine learning-based integrative procedure for constructing a consensus cell death-related signature risk score (CDRscore) model and validated by external data. The biofunctions including different expression analysis, immune cell infiltration, genomic mutations, enrichment analysis as well as cellchat analysis were compared between the high- and low- risk score groups categorized by this CDRscore model. Finally, samples from laryngeal squamous cell cancer (LSCC) were conducted by spatial transcriptomics (ST) to further validate the results of CDRscore model.

**Results:**

T cells from HNC patients manifested the highest levels of cell death while HPV infection attenuates malignant cell death based on single-cell atlas. CDMs are positively correlated with the tumor-cell stemness, immune-related score and T cells are infiltrated. A CDRscore model was established based on the transcription of ten cell death prognostic genes (*MRPL10*, *DDX19A*, *NDFIP1*, *PCMT1*, *HPRT1*, *SLC2A3*, *EFNB2*, *HK1*, *BTG3* and *MAP2K7*). It functions as an independent prognostic factor for overall survival in HNC and displays stable and powerful performance validated by GSE41613 and GSE65858 datasets. Patients in high CDRscore manifested worse overall survival, more active of epithelial mesenchymal transition, TGF-β-related pathways and hypoxia, higher transcription of T cell exhausted markers, and stronger *TP53* mutation. ST from LSCC showed that spots with high-risk scores were colocalized with TGF-β and the proliferating malignant cells, additionally, the risk scores have a negative correlation with TCR signaling but positive association with *LAG3* transcription.

**Conclusion:**

The CDRscore model could be utilized as a powerful prognostic indicator for HNC.

## Introduction

Head and neck cancer is the seventh most common malignancy globally. There are more than 890,000 new cases and 450,000 deaths annually according to the 2020 global cancer statistics (1, 2). HNC have several major subtypes like thyroid carcinoma (TH), hypopharyngeal carcinoma (HP), oropharyngeal carcinoma (OP), nasopharyngeal carcinoma (NP), laryngeal squamous cell cancer (LSCC), oral squamous cell carcinoma (OC) and nasopharyngeal carcinoma (NC) *etc*, which are discretely categorized based on their anatomical origination and location (3). Various treatment strategies such as surgery, radiation therapy (RT), chemotherapy (CT), and immunotherapy (IT), are available in clinic settings, unfortunately, most patients are frequently diagnosed at advanced stages, and the five-year survival rate is only ~40% after standard treatment (4). Therefore, it is important to identify novel strategies or biomarkers that potentially provide precise diagnosis and therapeutics for HNC.

Cell death is a fundamental physiological process that controls various physiological phenomena including growth, development, aging, and diseases. Different from accidental cell death (ACD), related cell death (RCD), also known as programmed cell death (PCD), can be triggered by specific signal transduction mechanisms and/or metabolic reprogramming (5, 6). In 2018, twelve different cell death modes (CDMs), including intrinsic apoptosis, extrinsic apoptosis, mitochondrial permeability transition-driven necrosis (mpt), necroptosis, ferroptosis, pyroptosis, parthanatos, entotic cell death, NETotic cell death, lysosome-dependent cell death, autophagy-dependent cell death, immunogenic cell death, cellular senescence, and mitotic catastrophe, were approved by the Nomenclature Committee on Cell Death (NCCD) (7). Recently, some additional types of CDMs like autosis, cuproptosis, anoikis, disulfidptosis, alkaliptosis, oxeiptosis, and mitotic cell death were also described (8). Zou Y et al. screened twelve PCD genes in tissues from triple-negative breast cancer and found that patients with high cell death index present poorer postoperative prognosis (9). Gao Y et al. suggested that autophagy, ferroptosis, pyroptosis and necroptosis have synergistic anti-tumor effects (10). Moreover, targeting mitochondrial apoptosis also increases the efficacy of natural killer (NK) cell-based immunotherapy (11). Interestingly, it seems that some forms of PCD, like necroptosis, pyroptosis, ferroptosis, NETosis and cuproptosis, might play essential roles in the invasion and metastasis of head and neck squamous cell carcinoma (12, 13). HPV-positive HNC is more sensitive to mitochondria-targeted treatment due to ferroptosis, therefore, inducing ferroptosis by dyclonine and paclitaxel (PTX) successfully prevent HNC recurrence after radiotherapy and/or chemotherapy (14). Although multiple cell death genes have been identified as diagnostic and/or prognostic biomarkers for some cancers, unfortunately, the comprehensive analysis of CDMs in HNC is unavailable till now.

The single-cell RNA sequencing (scRNA-seq) technique provides a transcriptomic atlas at high resolution for exploring cellular subpopulations and for dissecting specific molecules associated with disease progression (15), however, the original interaction information of cells-cells cannot be obtained due to the mechanical dissociation and enzymatic digestion of tissues. Recent advances in spatial transcriptomics (ST) have enabled acquisition of spatial information and transcriptome data simultaneously (16, 17). Because it provides high-quality genome-wide transcriptome data with intact two-dimensional positional information, ST has been successfully applied to analyze the spatial heterogeneity of human primary breast cancer (18), prostate cancer (19) and pancreatic ductal adenocarcinomas, *etc* (20). Therefore, ST can be used to decipher the characteristic genes or cell-cell communication that might trigger malignant transformation.

In this study, scRNA-seq data from five major subtypes of HNC were obtained from the GEO database. The distribution of eighteen different types of CDMs on these samples at single-cell level was investigated. Additionally, the cell death prognostic genes related to HNC were selected to establish a consensus cell death-related signature risk score (CDRscore) model using various machine learning algorithms. The biofunctions in patients with the high- and low- risk score that categorized by this CDRscore model were also compared. Finally, samples from LSCC were recruited to perform ST *in situ* to further validate the results predicted by our CDRscore model.

## Result

### The distribution of different CDMs in HNC based on scRNA-seq atlas

**Figure 1:**
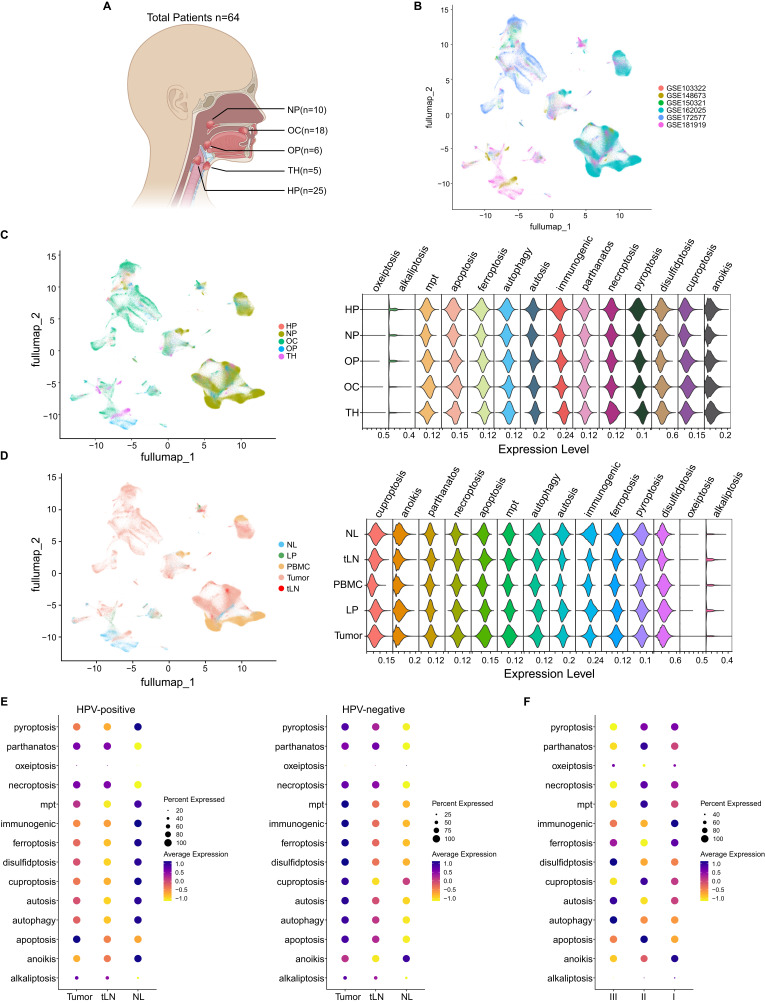
Deciphering the distribution of CDMs in HNC. **(A)** Schematic representing patients were recruited in this study. TH, thyroid carcinoma; HP, hypopharyngeal carcinoma; OP, oropharyngeal carcinoma; NP, nasopharyngeal carcinoma; OC, oral squamous cell carcinoma. **(B)** UMAP projection of 326178 cells aggregated from six different datasets. **(C)** UMAP projection of HNC subtypes aggregated (left) and the violin plot shows the scores of different CDMs in different HNC subtypes (right). **(D)** UMAP projection of different HNC tissues aggregated (left) and the violin plot shows the scores of different CDMs in different HNC tissues (right). **(E)** HNC patients were categorized into HPV-positive (left) and HPV-negative (right) groups and the distribution of different CDMs was shown. **(F)** The status of different CDMs in different disease stages was shown.

scRNA-seq data from the major five subtypes of HNC, including TH (n=5), OC (n= 18), HP (n= 25), OP (n= 6) and NP (n= 10) were collected from six different datasets (GSE103322, GSE148673, GSE150321, GSE162025, GSE172577 and GSE181919) (**Figures 1A, B**). These scRNA-seq data were further used to systematically analyze the distribution of eighteen different types of CDMs based on the transcription of characteristic genes (**Supplementary Table S1**). Fourteen kinds of CDMs were observed in these samples. It seems that disulfidptosis, necroptosis and pyroptosis might be the predominant CDMs in HP, whereas pyroptosis is the major CDM in NP. The levels of apoptosis, ferroptosis, autophagy and autosis were significantly upregulated in OP samples, whereas OC tissues manifested the highest levels of mpt, whereas TH cases showed extremely high levels of anoikis and apoptosis (**Figure 1C**).

We also compared the status of these fourteen kinds of CDMs in cells from normal larynx (NL, n=9), laryngeal leukoplakia (LP, n= 4), tumor tissues (n=61), PBMCs of HNC patients (n=10), and metastatic tumors in lymph nodes from HNC patients (tLN, n= 9). Remarkably, LP manifested extremely higher levels of ferroptosis and pyroptosis than other samples, conversely, NL samples present stronger levels autophagy, autosis, immunogenic cell death, ferroptosis and pyroptosis than tumor as well as tLN (**Figure 1D**). The infection of high-risk HPV, particularly HPV-16, normally deteriorates HNC carcinogenesis (21), therefore, we screened the samples with annotations of HPV infection status and categorized them into HPV-positive (n=7) and HPV-negative (n=16) groups. Surprised, the tLN samples from HPV-positive patients manifested extremely lower levels of cell death than NL controls, conversely, the tumor and tLN samples from HPV-negative patients showed significantly higher levels of cell death (**Figure 1E**). HNC are classified into three different stages according to the tumor-node-metastasis (TNM) systems (22), and patients in stage-I and -II manifest higher levels of necroptosis, cuproptosis and anoikis than that of cases in stage-III (**Figure 1F**), suggest that HNC manifest different levels of cell death and HPV infection prevent cell death in malignant cells.

### The status of CDMs in different cell subsets based on scRNA-seq atlas

**Figure 2:**
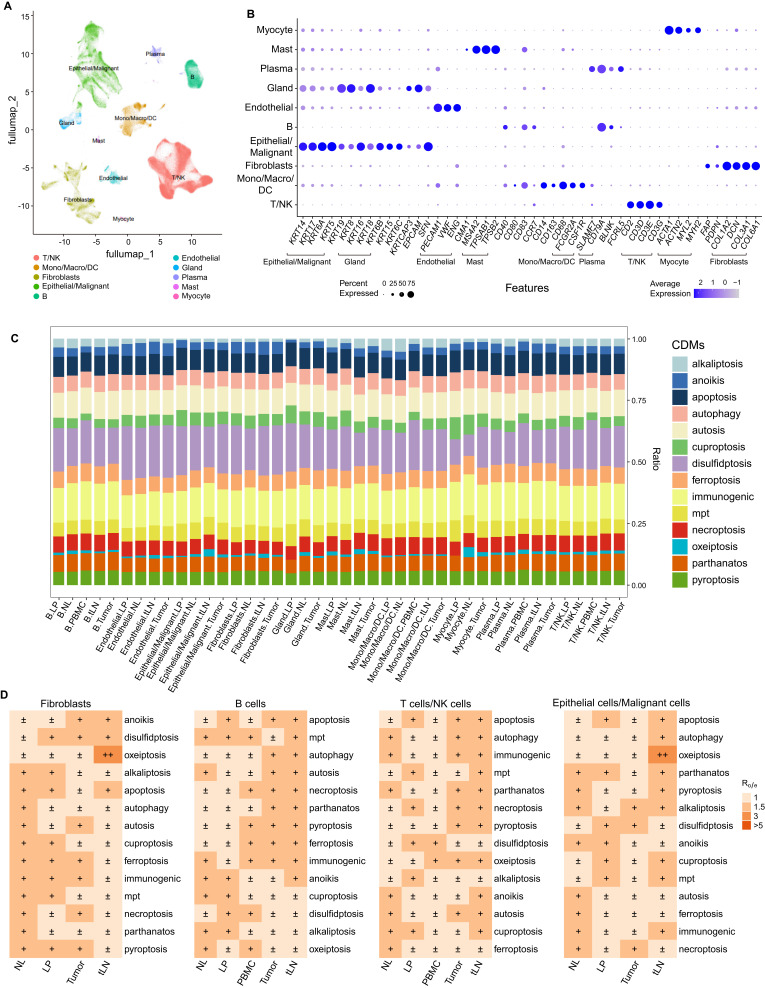
Depicting different CDMs in cell subsets based on scRNA-seq atlas. **(A)** UMAP projection of cell type annotation for HNC single-cell atlas. **(B)** Dot plot showed the typic marker expression of each subtype. **(C)** Stacked bar plot showed the proportion of CDMs in each subtype. **(D)** The Fibroblasts, B, T/NK and Epithelial/Malignant cells in different tissues from HNC, and the distributions of specific CDMs were showed.

The single cells from these HNC samples were further divided into ten distinct subsets by unsupervised clustering based on the expression of top variable feature genes (**Figures 2A, B**). We found that utosis, disulfidptosis, and immunogenic cell death are the three predominant CDMs across all cell subsets (**Figure 2C**). It seems that oxeiptosis is the major CDM in fibroblasts from tLN, and the immune cells (including B cells and NK/T cells) isolated from tumor and tLN tissues showed strong apoptosis, mpt, autophagy, autosis, necroptosis, parthanatos, pyroptosis, ferroptosis, immunogenic cell death and anoikis. Importantly, malignant cells/epithelial cells from tumor tissues manifested significantly lower levels of cell death (**Figure 2D**), demonstrating that immune cells have high levels of cell death.

Subsequently, we used monocle to predict the developmental trajectory of cells to assess the malignant degree of tumor cells. As the quasi-temporal sequence value continued to increase, the malignant degree of epithelial/malignant cells continued to increase (**Supplementary Figure S1A, B**). We observed that parthanatos and pyroptosis increased in malignant cells, while apoptosis and disulfidptosis decreased (**Supplementary Figures S1C-T**).

### T cells from tumor patients manifested the highest levels of cell death

**Figure 3:**
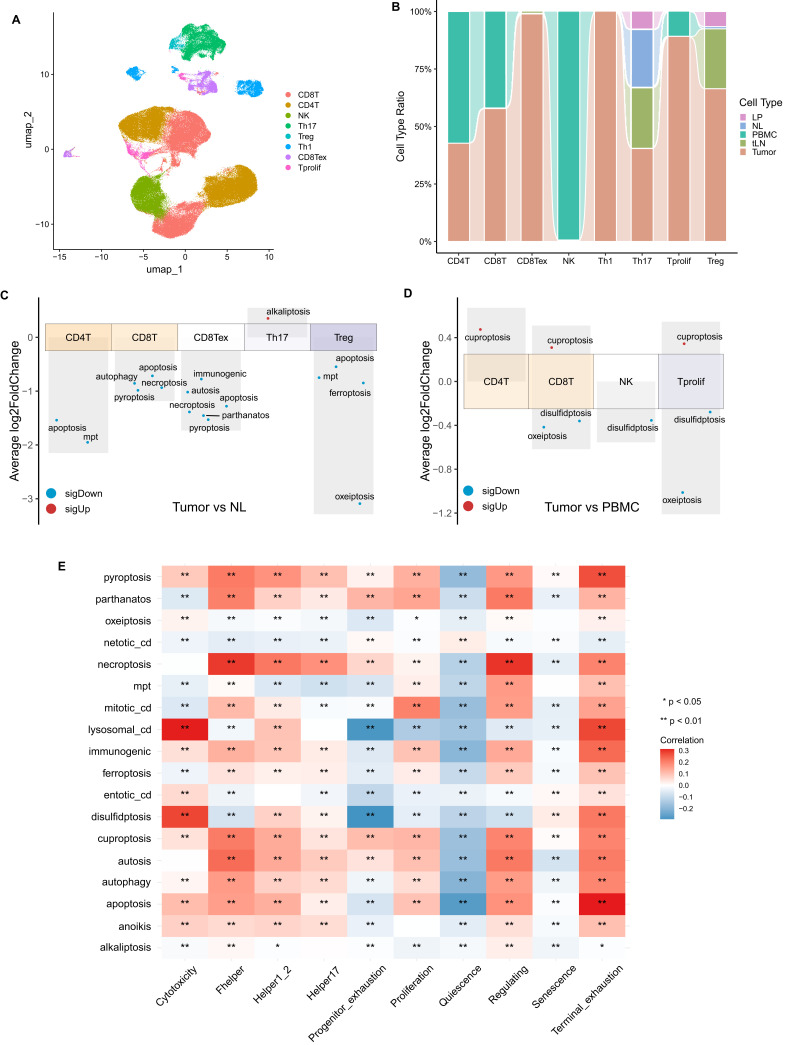
T cells from HNC patients manifested high levels of cell death. **(A)** UMAP projection of T/NK subtypes from HNC based on single-cell atlas. **(B)** bar plot showed the proportion of T/NK subtypes. Volcano plot showed the differences of cell death models in T/NK subtypes from tumor tissues vs. normal tissues **(C)**, and tumor tissues vs. patients’ PBMCs **(D)**. **(E)** The heatmap showed the correlations between T cell state and cell death in T/NK cells. *p<0.05, **p<0.01.

Tumor-infiltrating lymphocytes (TILs) are the main cells in tumor stromal tissues that carry out immunotherapy. TILs include T-helper cells (CD4^+^), cytotoxic T cells (CD8^+^), Foxp3^+^CD4^+^ regulatory T cells (Tregs), tumor-associated macrophages (TAMs), natural killer cells (NKCD57^+^), and myeloid-derived suppressor cells (MDSCs) *etc* (23). T cells were further subdivided into seven different subpopulations, including CD8^+^T, CD4^+^T, Th17, Treg, Th1, exhausted CD8^+^T cells (CD8Tex), and proliferating CD3^+^T cells (Tprolif), through automatic annotation (**Figure 3A**). Our data showed that CD8Tex, Treg, Th1, and Tprolif are majorly originated from tumor tissues, whereas CD4^+^T and NK cells are predominantly isolated from PBMCs (**Figure 3B**). The status of different CDMs was compared in CD4^+^ T, CD8^+^ T, CD8^+^ Tex, Th17 and Treg between tumor tissues and NL controls, results showed that Th17 cells from tumor tissues manifested dramatically higher levels of alkaliptosis, whereas CD4^+^T and Treg from tumor tissues presented a decreased in apoptosisa (**Figure 3C**). We further compared the status of CDMs between the circulating T cells derived from PBMCs and tumor-infiltrating T cells, and results showed that enhancement of cuproptosis is observed in CD4^+^ T, CD8^+^ T and Tprolif that derived from PBMCs of patients (**Figure 3D**). Subsequently, we evaluated the correlations between the state of T cells and cell death, and found that T cells in cytotoxicity manifested significantly high level of lysosomal_cd, and T cells in terminal exhaustion showed high levels of pyroptosis and apoptosis (**Figure 3E**) highlighting that T cells from tumor patients manifest higher level of cell death.

### T cells are more susceptibility to cell death validated by bulk-RNA sequencing data

**Figure 4:**
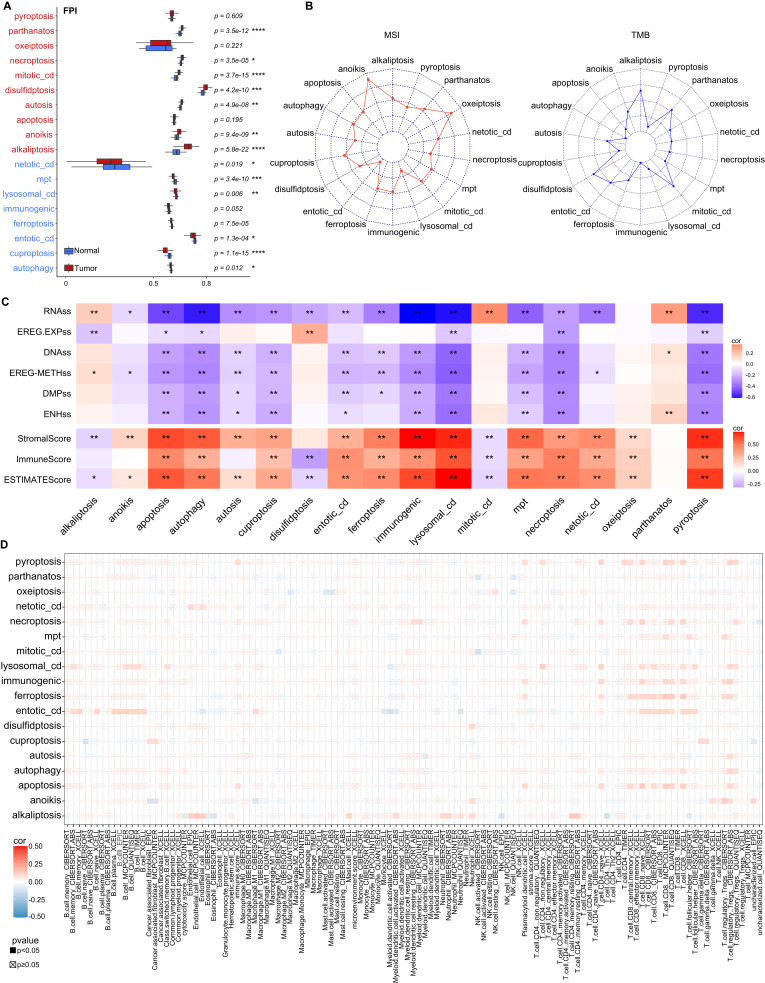
The distribution of different CDMs in HNC *via* Bulk RNA-seq atlas. **(A)** Box plot showed the different of CDMs between tumor tissues and normal tissues in all TCGA-HNSC bulk RNA sequencing atlas. **(B)** The correlations of different CDMs and MSI (left) as well as TMB (right). **(C)** The heatmap showed the correlations between immune score and different CDMs, and dots indicate statistically significant results **(D)** Dot plot showed correlations between immune cell infiltration and different CDMs. *p<0.05, **p<0.01, ***p<0.001 and ****p<0.0001.

We then collected bulk RNA-seq data of HNC from TCGA database to validate the distribution of different CDMs. Compared to NL controls, the tumor samples from HNC exhibit significantly higher levels of parthanatos, necroptosis, mitotic_cd, disulfidptosis, autosis, anoikis as well as alkaliptosis, whereas cuproptosis and mpt were reduced dramatically (**Figure 4A**).

The tumor mutation burden (TMB), microsatellite instability (MSI), and immune scores (IS) are often used as indicators to predict the response to immunotherapy (24), however, no associations were observed between the CDMs and MSI as well as TMB (**Figure 4B**). ESTIMATE algorithm could calculate tumor microenvironment (TME) score by expression profiles data, such as stromal score, immunity score, and tumor purity (25). We found that many CDMs including apoptosis, autophagy, immunogenic cell death, lysosomal_cd and pyroptosis, are positively correlated with StomalScore, ImmuneScore and ESTIMATEscore, whereas they are negatively associated with RNA stemness score (RNAss) (**Figure 4C**). Further investigations showed that apoptosis, autophagy, immunogenic cell death, lysosomal_cd and pyroptosis are restricted in T cells, especially CD8^+^ T cells in tumor tissues (**Figure 4D**), confirming T cells from HNC patients are more susceptibility to cell death.

### Integrative construction of a consensus cell death-related signature

**Figure 5:**
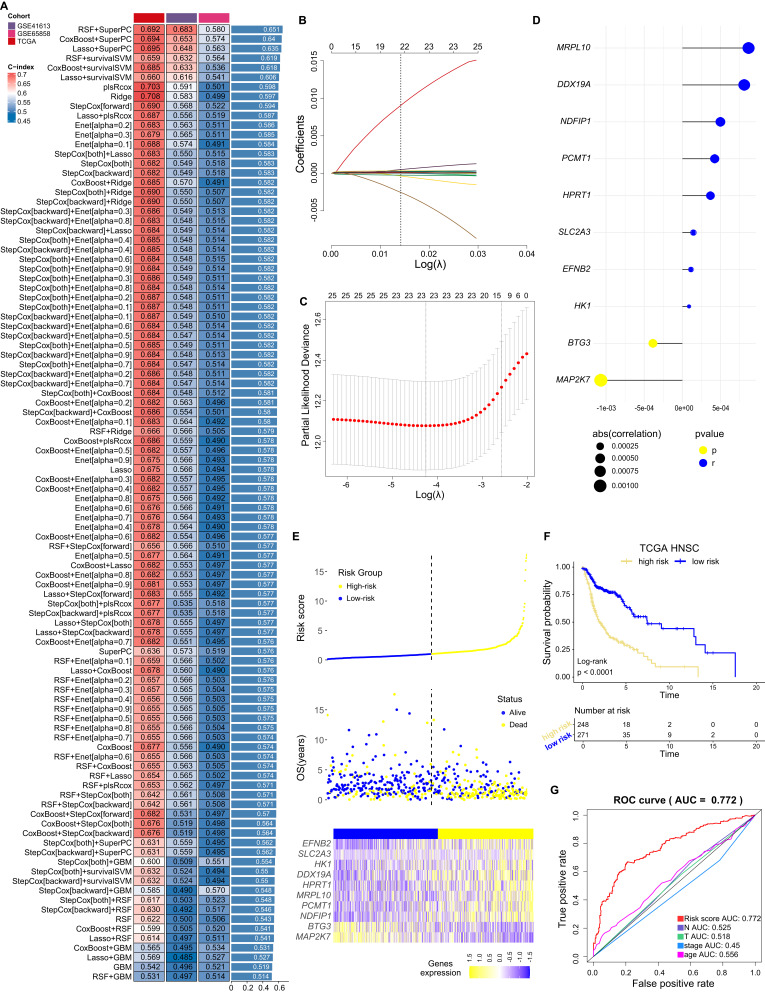
Integrative construction of a consensus CDR signature. **(A)** A total of 100 kinds of prediction models *via* LOOCV framework and further calculated the C-index of each model across all validation datasets. **(B)** LASSO regression analysis of the cell death related genes selected by RSF and superPC. **(C)** The plot showed the determination of the optima with log(λ) values on the abscissa for LASSO regression analysis. **(D)** Ten cell death related genes finally obtained in stepwise Cox regression. **(E)** Distribution of risk score, patients’ survival status and the transcription of indicated genes in high/low risk group from TCGA-HNSC based on our RCD signature. **(F)** Kaplan-Meier curves of OS according to our CDR signature in TCGA-HNSC. **(G)** ROC curves for our CDR signature with risk score and clinical data.

To explore the clinical application value of cell death, univariate Cox analysis was selected to identify 154 prognostic genes from the expression profiles of these eighteen CDMs (**Supplementary Table S2**). These prognostic genes were subjected to our machine learning-based integrative procedure to develop a consensus cell death-related (CDR) signature. In the TCGA-HNSC dataset, we fitted 100 kinds of prediction models through the LOOCV framework to build model and further calculated the C-index of each model across three validation datasets (GSE41613, GSE65858 and TCGA). Interestingly, the optimal model was a combination of RSF and superPC (direction=both) with the highest average C-index (0.651), and this combination model had a leading C-index in all validation datasets (**Figure 5A**). Furthermore, the Lasso regression was used to reduce variables from the optimal model (**Figure 5B**), and the optimal λ was obtained when the partial likelihood deviance reached the minimum value based on the LOOCV framework (**Figure 5C**). Twenty-five cell death associated genes with nonzero Lasso coefficients were subjected to stepwise Cox proportional hazards regression, which identified a final set of 10 genes (*MRPL10*, *DDX19A*, *NDFIP1*, *PCMT1*, *HPRT1*, *SLC2A3*, *EFNB2*, *HK1*, *BTG3* and *MAP2K7*) to build the final CDR signature (risk score = (4.985107*10^-4^)*NDFIP1_EXP_ + (-1.068404*10^-03^)*MAP2K7_EXP_ + (1.441470*10^-04^)*SLC2A3_EXP_ + (1.123539*10^-04^)*EFNB2_EXP_ + (3.678527*10^-04^)*HPRT1_EXP_ + (8.106291*10^-04^)*DDX19A_EXP_ + (-3.859801*10^-04^)*BTG3_EXP_ +(8.725573*10^-05^)*HK1_EXP_ + (4.235660*10^-04^)*PCMT1_EXP_ + (8.672*10^-04^)*MRPL10_EXP_, **Figure 5D**). We established a CDR signature based on these ten genes and calculated the risk score for each patient from TCGA-HNSC dataset, and patients were then categorized into high- and low- risk score groups based on the optimal cut-off value which determined by the *survminer* package. The risk score, patient status (alive or death), overall survival (OS), and the transcription of these 10 genes in various clinical features were described (**Figure 5E**). Interestingly, patients in the high-risk score group had significantly dismal OS relative to the low-risk score group (*p*<0.0001, **Figure 5F**). The area under curve (AUC) value of receiver operating characteristic (ROC) curve is 0.772, higher than others clinical classifications (**Figure 5G**). Collectively, we established a cell death related risk score (CDRscore) model based on the CDR signature and this model is more accurate than other clinical information in predicting the prognosis of HNC.

Data from two other datasets (GSE65858 and GSE41613) were also recruited to validate the clinical values of our CDRscore model, and results confirmed that this CDRscore model well distinguished the patients’ OS due to cases in the high-risk score group had significantly dismal OS relative to the low-risk score group (**Supplementary Figures S2A, B**). A nomogram consisting of a CDRscore model and various clinicopathological features were established to accurately forecast the survival of HNC patients (**Supplementary Figure S2C**). The calibration curve was used to verify the validity and accuracy of the nomogram for the predictive OS probability of 3- and 5-year in TCGA-HNSC patients (**Supplementary Figure S2D**). The DCA curves showed that the CDRscore model was more beneficial for predicting the outcome of HNC patients than any single prognostic factor (**Supplementary Figure S2E**), indicating our CDRscore model has clinical utility for predicting HNC prognosis.

### Different physiological characteristics in patients with high- and low- risk CDRscore

**Figure 6:**
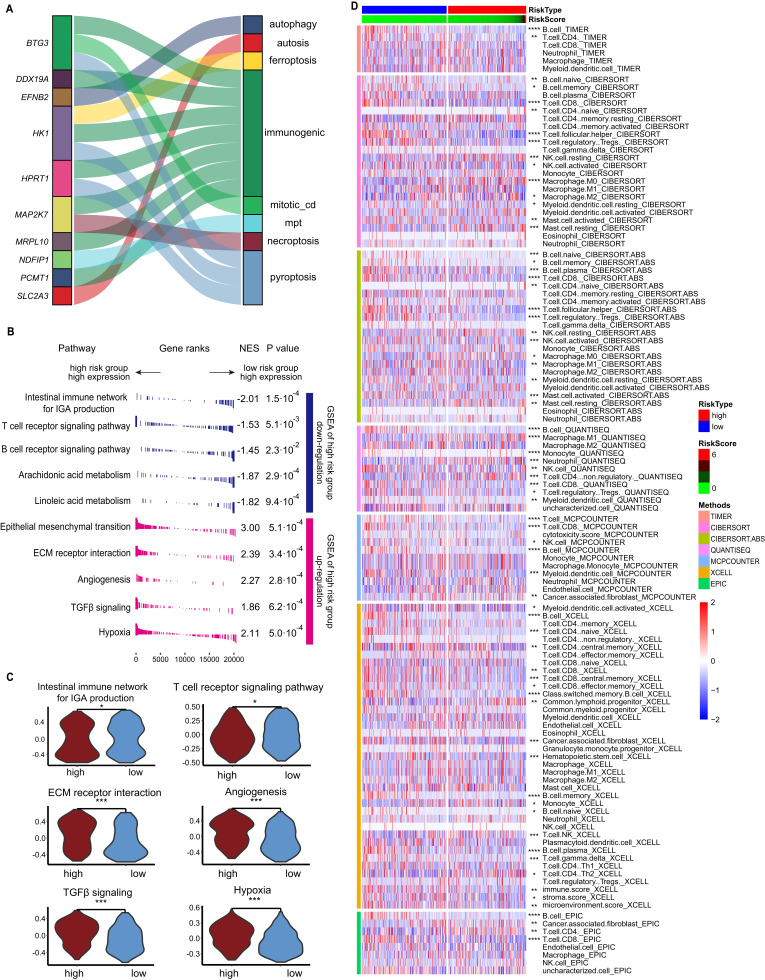
Different biofunctions between the high- and low- risk score groups classified by the CDRscore model. **(A)** Graphical summary of the ten signature genes and the CDMs. **(B)** GSEA of the upregulation and downregulation in high-risk score group. **(C)** Box plot showed the different GSVA results between high- and low-risk score groups. **(D)** Heatmap showed the different immune cell infiltration based on seven different analytic method between high- and low-risk group. *p<0.05, **p<0.01, ***p<0.001 and ****p<0.0001.

The cell death-associated genes involved in construction of our CDRscore model are associations with autophagy, autosis, ferroptosis, immunogenic, mitotic_cd, mpt, necroptosis and pyroptosis (**Figure 6A**). We then compared the transcriptional alterations in patients with high- and low- risk scores classified by our CDRscore model, and Gene Set Enrichment Analysis (GSEA) showed that most immune response associated pathways, such as T/B cell receptor signaling pathway and Intestinal immune network for IgA production, were significantly down-regulated in patients with high-risk CDRscore, nevertheless, the tumor development related pathway, including epithelial mesenchymal transition, ECM receptor interaction, angiogenesis, TGF-β signaling and hypoxia, were significantly up-regulated (**Figure 6B**). These results were also supported by Gene Set Variation Analysis (GSVA) (**Figure 6C**), suggesting that the TME from the high- and low-risk CDRscore patients are completely difference. Subsequently, we used seven different mainstream methods (TIMER, CIBERSORT, CIBERSORT.ABS, QUANTISEQ, MCPCOUNTER, XCELL and EPIC) to analyze the differences of immune cell infiltration between high- and low- risk score groups, results showed that the infiltration of macrophages (M0) and neutrophils were increased significantly in the high-risk score group, while the infiltration of B cells, macrophages (M2) and CD8^+^ T cells was decreased dramatically compared to low- risk score group (**Figure 6D**).

Large-scale whole-exome sequencing reveal that the genomic mutations of many genes, including *TP53*, *CDKN2A*, *PTEN*, *PIK3CA*, *HRAS*, *NOTCH1*, *IRF6*, and *TP63*, are a major driver of HNC carcinogenesis (26, 27). We also compared the counts of genomic mutations, and no significant differences were observed between these high- and low-risk score groups, neither synonymous nor non-synonymous mutation (**Supplementary Figure S3A**). The top ten genes with high-frequency mutations in the high-risk CDRscore group (n=238) and low-risk score group (n=246) was also compared (**Supplementary Figure S3B**). Interestingly, the mutation rate of *TP53* in the high-risk score group is significantly higher than that in low-risk score group, conversely, the low-risk score group has a significant increase in *NSD1* mutation frequency than that in the high-risk score group (**Supplementary Figure S3C**), demonstrating patients in high- and low- risk score groups have different genomic mutations.

### Explore the impact of CDRscore model at single-cell level based on scRNA-seq

We explored the distribution of these ten genes associated to our CDRscore model at single-cell level (**Supplementary Figure S4A**). We found that most genes were expression in malignant cells, such as *NDFIP1*, *MRPL10*, *HPRT1*, *BTG3*, *PCMT1* and *HK1*. It seems that the distributions of *NDFIP1*, *PCMT1* and *SLC2A3* are broadly and across all detected cell subsets. Comparative analysis showed that genes such as *BTG3*, *PCMT1* and *HK1* are gradually increasing in epithelial/malignant cells from tumor tissues compared to these cells from normal tissues (**Supplementary Figure S4B**).

**Figure 7:**
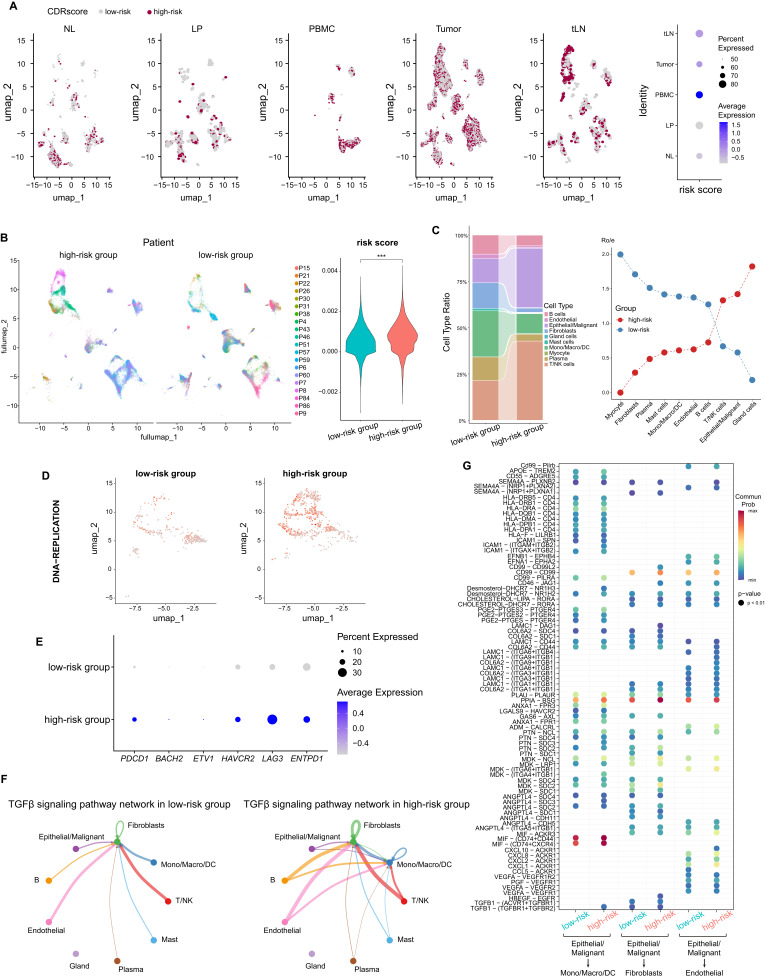
Explore the impact of CDRscore risk model at single-cell level based on scRNA-seq. **(A)** UMAP projection of risk score in different tissues from HNC (left), and dot plot showed the average and percent risk score in different tissues (right). **(B)** UMAP projection of different patients from GSE181919 based on high- and low- risk scores (left), and the violin plot shows the difference in risk score between high- and low- risk score groups (right). **(C)** The stacked histogram shows the differences in cell composition between high- and low-risk groups (left), and the dot plot shows the difference in Ro/e values between high- and low-risk groups (right). **(D)** UMAP projection of the score of DNA replication pathway in malignant cells of patients from high and low-risk groups. The darker the color, the higher the score,which indicating stronger activity of the DNA replication pathway. **(E)** Dot plot showed the transcription of T cell exhausted-related genes between high- and low-risk score groups. **(F)** Communication quantities of TGF-βsignaling pathway network among each cell types in high- and low-risk score groups. **(G)** Different LRIs in high- and low-risk groups. ***p<0.001.

We then compared the distribution of risk scores in various tissues, and results showed that NL presented lowest level of risk score, but the risk scores were elevated significantly in tumor, PBMCs and tLN (**Figure 7A**). Patients from the GSE181919 dataset were also categorized into the high- and low-risk score groups based on our CDRscore model, and the risk scores of these two groups showed significant difference (**Figure 7B**). Moreover, the frequency of malignant cells and T/NK cell infiltration in the high-risk score group were significantly higher than those from the low-risk score group (**Figure 7C**). The proliferation ability of malignant cells (indicated by DNA replication) (**Figure 7D**), and the transcription of exhausted-related marker genes (*PDCD1*, *BACH2*, *ETV1*, *HAVCR2*, *LAG3* and *ENTPD1*) on T cells (**Figure 7E**) in the high-risk score group was significantly higher than that in the low-risk score group. Previous work have demonstrated that the expression of exhausted-related marker genes and malignant cell proliferation is induced by TGF-β signaling (28), and analysis of the TGF-β signaling pathway network indicated that patients with high-risk scores presented a higher activity of TGF-β signaling (**Figure 7F**). Especially increased binding of TGF-β related ligand-receptors such as ACVR1- TGFBR1 ligand-receptors pairs, indicating that although the infiltration of T cells in the high-risk group increased, most of the T cells were depleted and did not exert anti-tumor functions (**Figure 7G**).

### Verification of our CDRscore model *via* ST *in situ*

**Figure 8:**
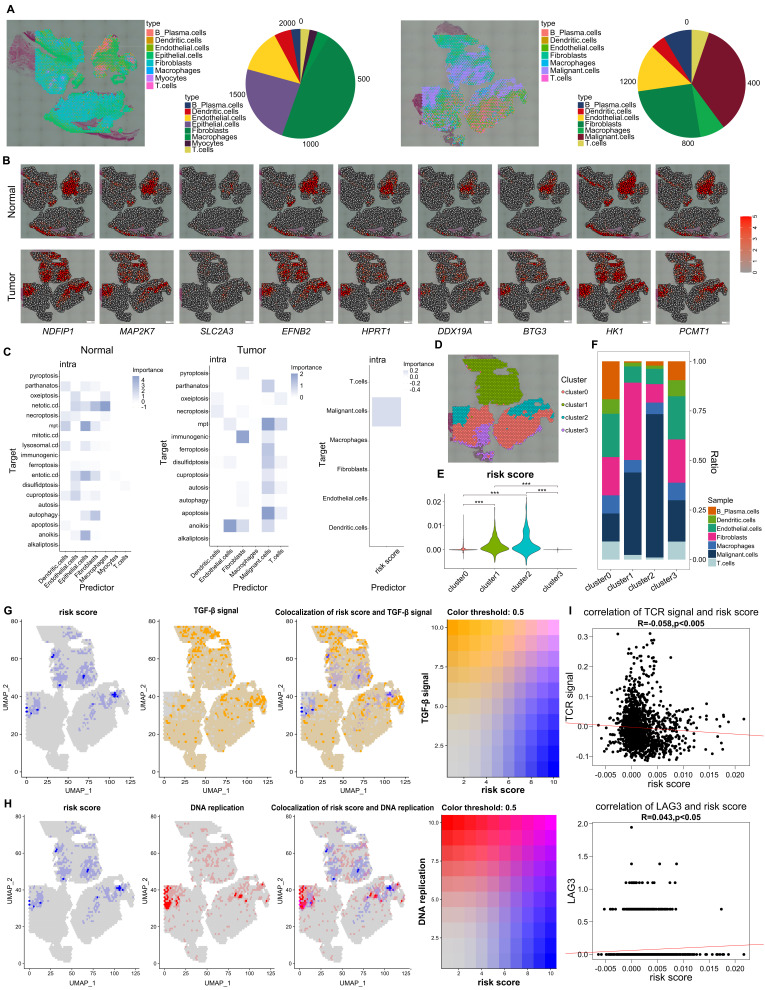
Verification of our CDRscore model *via* ST. **(A)** Deconvoluted ST images of normal samples (left) and tumor samples (right) from LSCC, each spot included a pie chart and showed potential cell composition. The pie chart on the right showed the total proportion of cells from two samples, respectively. **(B)** The ST images showed the transcription of genes from CDR signature in normal and tumor tissues. **(C)** The median importance of different cell death models in the predicting cell subsets of normal (left), and tumor tissues (middle), and high risk-score was seen in malignant cells (right). **(D)** ST images showed four different clusters in tumor sample. **(E)** Statistic analysis of risk score among four different cluster. **(F)** the proportion of different cell subtypes across clusters. **(G)** ST images showed the colocalization between risk score and TGF-β signaling **(G)** and DNA replication **(H)**. **(I)** The correlations between risk score and TCR signaling pathway (up panel) and the transcription of *LAG3* (down panel). ***p<0.0001.

To verify our above results, we then performed the ST from LSCC patients (n= 2) and normal larynx control samples (n=1) based on 10 x Genomics Visium. The diameter of each spot was 55μm (capturing 8 ~ 20 cells) of this 10 x Genomics Visium, and each tissue section within the capture area (6.5 mm x 6.5 mm) contained up to 4900 spots. We annotated cell types of ST by deconvolution, and normal larynx tissues showed high frequencies of epithelial cells and fibroblasts, whereas, malignant cells, T cells and B cells were increased significantly in LSCC samples (**Figure 8A**). The transcription of genes associated to our CDRscore model were increased significantly in LSCC samples, except that *MRPL10* was not detected in ST (**Figure 8B**). It seems that LSCC and NL manifested different kinds and levels of cell death, and malignant cells manifested high levels of mpt and apoptosis, while immunogenic is the main cell death in fibroblasts (**Figure 8C**). LSCC samples with high-risk score based on our CDRscore model was mainly enrichment in malignant cells (**Figure 8D**). After dimension reduction and clustering of tumor samples, cluster1 and cluster2, which mainly contain malignant cells, presented significantly higher risk score than other clusters (**Figure 8E**). We also found the spots with high-risk score are colocalization with malignant cell proliferation (DNA replication) and TGF-β signaling. Most important, both the TGF-β signaling and malignant cell proliferation highly overlap with spots of high-risk scores at the interface between malignant cells and immune cells (**Figure 8F**). Additionally, the high-risk score is negatively associated with genes associated with T cell receptor (TCR) signaling pathway but is positively correlated with *LAG3* gene expression (**Figure 8G**).

## Discussion

HNC is the 7^th^ prevalent type of cancer that occur in the mucosal surfaces of oral cavity, nasopharynx, oropharynx, hypopharynx, and larynx. Tobacco, alcohol, betel nuts, and human HPV infection are the main risk factors (22). Surgery, radiation, chemotherapy as well as immunotherapy, have been selected for advanced HNC treatment, unfortunately, the efficiency of these methods is limitation, probably due to various genetic and epigenetic alterations (29). It is generally accepted that cancer cells are resistant to apoptosis, and induction of tumor cell death is a novel treatment strategy (30). A cohort of classical forms of CDMs have been studied in different kinds of tumor, and deciphering novel CDMs in the regression of HNC might supply some promising strategies for therapy. Here, scRNA-seq data from sixty-four cases of HNC were collected in six different datasets and used for pan-cell death analysis at single-cell level, results showed that T cells rather malignant cells manifest higher levels of cell death. Previous work have showed that the immunotherapy based on immune checkpoint inhibitors (ICIs) sometimes is limitation probably is due to the absence of TILs (31), our results support this conclusion and further illustrated that cell death, including alkaliptosis and cuproptosis, might involve in inducing T cell deletion within HNC tissues.

Recently, NCCD established guidelines encompassing morphological and biological aspects of eighteen different types of CDMs (32), some kinds of CDMs are controlled by specific signal transduction pathways, including p53 signalling, KRAS signalling, NOTCH signalling, hypoxia signalling, and metabolic reprogramming (6). The potential roles of these different kinds of CDMs in carcinogenesis, including HNC, were also analyzed. For example, Li Y et al., reported that necroptosis is an independent prognostic marker for HNC patient’s OS and progression-free survival (33). Patients of HNC with Caspase-8 mutation are sensitive to necroptosis signals and manifest better clinical outcomes (34). Numerous necroptotic drugs like Etoposide, Shikonin, TRAIL, staurosporine, taxol, 5-fluorouracil (5-FU), sorafenib, and camptothecin, have been used for the treatment of cancers including HNC (35). We here showed that CD8Tex cells in tumor tissues manifested extremely lower levels of necroptosis (**Figure 3C**), suggesting that deletion of CD8Tex cells *via* targeting the necroptotic pathway seems to be a potential therapeutic strategy for HNC.

It seems that higher ferroptosis potential index (FPI) is significantly associated with worse OS in HNC patients (36). SLC7A11, a key regulator of ferroptosis which transports extracellular cystine into cells for glutathione biosynthesis, is highly expressed in HNC, and reduction in ferroptosis through overexpression of *SLC7A11* has been found to be positively modulated by lymph node metastasis (37, 38). Importantly, some natural compounds like Artesunate, exhibits a specific ferroptosis effect on HNC cells through the upregulation of lipid ROS generation and the downregulation of cellular glutathione (GSH) levels (39), suggesting that therapies associated with HNC can be expected to focus on the regulation of ferroptosis-mediated cell death. We here showed that HPV infected malignant cells and Treg in tumor tissues present extremely low levels of ferroptosis (**Figure 1C**, **3C**), indicating that ferroptosis might participate into the development of HNC.

Cuproptosis has been recognized as a novel form of PCD that has been confirmed to promote the occurrence and development of tumors. Based on exploration through bioinformatics, researchers have established that cuproptosis-related lncRNA has an impact on prognosis in HNC (40). Zhang S et al. elucidated the relationship between cuproptosis and the immune microenvironment in HNC, highlighting the fact that cuproptosis metabolism may be a possible predictive biomarker for HNC treatment (41). Additionally, OSCC cell metastasis is closely associated with cuproptosis with high expression of AFOC-DEGs (42). We here showed that the effective T cells, including CD4^+^T, CD8^+^T and Tprolif in tumor tissues have higher levels of cuproptosis than T cells from PBMCs of tumor patients (**Figure 3C**), illustrating that the reduction of effective T cells within tumor tissues might be caused by cuproptosis.

Machine Learning (ML) algorithms are artificial intelligence (AI)-driven algorithms that can profoundly impact biomedical research, personalized and precision medicine (43). By analyzing genomics, pathomics, imaging, and other biological data with computers, mathematical modeling, and applying it to clinical and scientific research, ML is a method for discovering novel things on patients. For example, Liu W et al. developed a random forest ML model that accurately predicts the risk of bone metastasis in patients with thyroid cancer (44). Zhu J et al., established an optimal XGBoost model to predict the risk of central lymph node metastasis in patients with papillary thyroid carcinoma (45). Moreover, ML algorithm models are better than traditional methods in predicting early-stage colorectal cancer lymph node metastasis (46). Recently, Liu Z et al., have developed a MI-based integrative procedure for constructing a consensus immune-related lncRNA signature IRLS, which is an independent risk factor for overall survival and displays stable and powerful performance (47). Here some cell death-associated prognostic genes were subjected to ML-based integrative procedure to develop a consensus CDR signature, we fitted 100 kinds of prediction models through the LOOCV framework to build model and further calculated the C-index of each model across three validation datasets (GSE41613, GSE65858 and TCGA). Interestingly, the optimal model was a combination of RSF and superPC (**Figure 5A**). Finally, a CDRscore model was established based on the transcription of ten cell death associated-genes (*MRPL10*, *DDX19A*, *NDFIP1*, *PCMT1*, *HPRT1*, *SLC2A3*, *EFNB2*, *HK1*, *BTG3* and *MAP2K7*), univariate and multivariate COX results indicated that CDRscore was an independent prognostic factor in HNC, patients in high CDRscore manifested worse survival rates, less immune cell infiltration, more active of epithelial mesenchymal transition, TGF-β-related pathways and hypoxia, higher transcription of T cell exhausted markers, and stronger *TP53* mutation. The TNM stages are conventional tools for evaluating clinical outcomes and treatment decisions for HNC. Additionally, some biomarkers, including TMB, microsatellite state; and *TP53*, *CDKN2A*, *PTEN*, *PIK3CA*, *HRAS*, *NOTCH1*, *IRF6*, or *TP63* mutation are also significantly correlated with the clinical strategies and outcomes (26, 27). Notably, our signature worked independently of these factors and had significantly superior performance in predicting prognosis according to the C-index assessment (**Figure 5F**).

Presently, stRNA-seq has been widely applied to investigate tumor heterogeneity due to this technique enables transcriptomic data to be acquired from intact tissue sections and provides spatial distribution information (48). stRNA-seq remedies the disadvantage of scRNA-seq, whose data lack spatially resolved information (49). For examples, Thrane K et al. found there is a unique tumor microenvironment near the tumor regions where multiple tumor-related signaling pathways were activated by applied stRNA-seq to decipher lymph node biopsies from melanoma (50). Yoosuf N et al., demonstrated that stRNA-seq can effectively enhance invasive ductal carcinoma diagnostic accuracy by stRNA-seq (51). Moncada R et al. observed that fibroblasts are enriched in tumor regions rather than in the matrix of sections from pancreatic cancer patients based on the combination of scRNA-seq and stRNA-seq data (20). We here also deciphered the spatial distributions between TGF-β, malignant cells and high-score of clusters LSCC samples, and ST from LSCC showed that clusters with high-risk scores were colocalized with TGF-β and the proliferating malignant cells, additionally, the risk scores have a negative correlation with TCR signaling but positive association with *LAG3* transcription (**Figure 8**), illustrating that data from ST validate our CDRscore model.

In summary, based on a multitude of bioinformatics and ML algorithms, we developed a stable and powerful CDRscore model for assessing the prognosis of HNC. This CDRscore model is a promising tool to optimize decision-making and surveillance protocols for individual HNC patients.

## Materials and methods

### Data acquisition

We collected Bulk RNA-seq and scRNA-seq data from GEO database (https://www.ncbi.nlm.nih.gov/geo/) and TCGA (https://portal.gdc.cancer.gov/). For bulk RNA-seq analysis, we obtained TCGA-HNSC datasets containing RNA-seq expression matrix, clinical information, and masked annotated somatic mutation from TCGA. Additionally,GSE65858 dataset, which contains 270 HNC samples (52) and GSE41613 dataset,which includes 97 OSCC samples (53) along with their clinical information, were downloaded from the GEO database. For scRNA-seq analysis, the following datasets were downloaded from the GEO database: GSE103322 (containing primary cancer and metastatic tumors in lymph nodes from oral cavity tumors) (54), GSE148673 (tumor tissues from anaplastic thyroid cancer) (55), GSE150321 (tumor tissues from laryngeal squamous cell carcinoma) (56), GSE162025 (tumor-blood pairs from nasopharyngeal carcinoma) (57), GSE172577 (tumor tissues from oral squamous cell carcinoma) (58) and GSE181919 (comprising normal tissue(NL), precancerous leukoplakia (LP), primary cancer(CA) and metastatic tumors in the lymph nodes) (59).

### scRNA-seq analysis

In our study, we processed a total of 326178 single cells utilizing data from six GEO datasets, which included GSE103322, GSE148673, GSE150321, GSE162025, GSE172577, and GSE181919. These datasets encompassed various types of tumor tissues and normal tissues, allowing us to perform comprehensive scRNA-seq analysis. Seurat (V5.0.3) was used to perform scRNA-seq analysis. First, Low quality cells (<500 genes/cell, >5% mitochondrial genes or a log10 (UMI *per* gene) < 3) were excluded,and then potential doublets were identified and excluded using scDblFinder (V1.16). Then, the expression matrix was normalized and scaled with default settings using Seurat.To reduce the computational load, sketch-based analysis was used to reduce dimensionality through principal component analysis (PCA) and UMAP.Unsupervised cluster analysis was then applied to identify clusters, and the results were subsequently applied to the full dataset.Marker genes within each cluster were pinpointed using the FindAllMarkers function from Seurat,with logFC > 0.2 and adjusted *p* -value  < 0.05. Then, clusters were annotated based on canonical maker genes from previously published studies.

### Enrichment analysis

To explore the potential biological functions of differentially expressed genes, Gene Ontology (GO) functions and Kyoto Encyclopedia of Genes and Genomes (KEGG) pathway enrichment analyses were performed using clusterProfiler(V4.7.1.003) (60). ClusterProfiler was also utilized to identify highly relevant KEGG and HALLMARK pathways differentiating the high-risk and low-risk subgroups through Gene Set Enrichment Analysis (GSEA) from the Molecular Signatures Database (MSigDB, http://software.broadinstitute.org/gsea/msigdb/).

### T cell state assessed

Tcellsi(v0.1.0) (T cell state identifier) was used to assess eight different states of T cells, including quiescence, regulating, proliferation, helper, cytotoxicity, progenitor, exhaustion, terminal exhaustion and senescence, based on the expression matrix of HNC scRNA-seq atlas.

### Pseudo-time analysis

Monocle2(V 2.24.0) was used to infer pseudo-time progression based on the expression matrix of malignant cells. High variable genes was used to filter data and reduce dimension (61).

### Construction of CDRscore signature

To develop a consensus cell death model with high accuracy and stability performance, we integrated 10 machine learning algorithms and generated 100 algorithm combinations. The integrative algorithms included random survival forest (RSF), elastic network (Enet), Lasso, Ridge, stepwise Cox, CoxBoost, partial least squares regression for Cox (plsRcox), supervised principal components (SuperPC), generalized boosted regression modeling (GBM), and survival support vector machine (survival-SVM). First, univariate Cox regression was used to identify prognostic genes based on the TCGA-HNSC cohort. Then, 100 algorithm combinations were performed on the prognostic genes to fit prediction models based on the leave-one-out cross-validation (LOOCV) framework within the TCGA-HNSC cohort and validated on GSE65858 and GSE41613 datasets. For each model, the Harrell’s concordance index (C-index) was calculated across all validation datasets, and the model with the highest average C-index was considered optimal. Thereafter, the feature variables selected for the optimal model were further reduced using LASSO regression, and a multi-factor stepwise Cox regression analysis was employed to finalize the model.

### GSVA

GSVA(v1.46.0) is an unsupervised and non-parametric technique utilized to evaluate gene set enrichment in transcriptomes. GSVA reallocates gene-level modifications to pathway-level modifications by integrating the scoring of gene sets of interest, thereby inferring the biological functions of the samples. In this study, we retrieve gene collections from the MsigDB (v7.0) and apply the GSVA algorithm to assess each collection comprehensively, to examine potential alterations in the biological functions of diverse samples.

### Immune infiltration analysis

We utilized the TIMER 2.0 (http://timer.comp-genomics.org/timer/) to analysis immune infiltration of TCGA-HNSC. In addition, other immune infiltration analyses results including CIBERSORT, QUANTISEQ, MCPCOUNTER,XCELL, EPIC were download from TIMER 2.0 (62).

### Cell-cell interaction inference

To analyze intercellular communication within our integrated cell database, we employed CellChat (V1.6.1, http://www.cellchat.org/) (63), using default parameters, to infer potential signaling interactions between cells based on a predefined database of ligand-receptor pairs. Special attention was given to the interactions between various Endothelial/Malignant cells and immune cells.

### Nomogram and calibration

To assess the prognostic value of the risk score over time in the entire TCGA dataset, we performed ROC analysis. Additionally, we investigated the role of the risk score in different clinical subgroups, including age, stage, T, N and gender. To provide a comprehensive predictive tool, we constructed a nomogram using multivariate Cox regression analysis, which integrated the risk score along with clinical information. Furthermore, we employed calibration curves to evaluate the accuracy of the constructed nomogram.

### LSCC samples collection

Two LSCC tissue samples and a normal tissue sample were collected. All clinical data were derived from surgical and pathological reports. Unless otherwise specified, all patients received standard postoperative care (SOC). The excised tissues were fixed in formalin and embedded in paraffin (FFPE), and pathological classifications were confirmed by three pathologists using Hematoxylin and Eosin (H&E) staining. All surgical specimens and clinical data were obtained after obtaining written informed consent from the patients. Clinical information was de-identified and used in accordance with the Institutional Review Board of the 989th Hospital of the People’s Liberation Army (REB: 2024-141).

### ST sequencing

This experiment are conducted by the Visium Technology Platform of 10x Genomics company. The reagents and consumables in the experiment are provided by this platform, and the specific product numbers can be found at www.10xgenomics.com/products/spatial-gene-expression.

### Slide preparation

This method has been described previously with slightly difference (64). Briefly, the Visium Spatial Gene Expression Slide (from Visium Spatial Gene Expression Slide Kit, 10x Genomics, PN-1000185) includes four capture areas (6.5 mm by 6.5 mm), each defined by a fiducial frame (fiducial frame + capture area is 8 mm by 8 mm). The capture area has ~5000 gene expression spots, each spot with primers that include Illumina TruSeq Read 1 (partial read 1 sequencing primer), 16–nucleotide (nt) spatial barcode (all primers in a specific spot share the same spatial barcode), 12-nt UMI; 30-nt poly(dT) sequence (captures polyadenylated mRNA for cDNA synthesis).

### RNA integrity number

We use RNeasy Mini Kit (QIAGEN, catalog no. 74104) to test the integrity of RNA. After taking 10 slices of 10-mm-thick cryosections, RNA was extracted and analyzed by RNeasy Mini Kit immediately. An RNA integrity number of ≥7 is qualified.

### Tissue fixation, staining, and imaging

Tissue sections on the Visium Slide (from Visium Slide Kit) were fixed using methanol (MilliporeSigma) by incubating 30 min at -20°C. For tissue staining, sections were incubated in isopropanol (MilliporeSigma) for 1 min, in hematoxylin (Agilent) for 7 min, in Bluing Buffer (Agilent) for 2 min, and in Eosin Mix (MilliporeSigma) for 1 min at room temperature. Last, slides were incubated for 5 min at 37°C in the Thermocycler Adaptor (10x Genomics, PN-3000380). Then, the stained tissue sections are imaged.

### Tissue permeabilization and reverse transcription

For tissue permeabilization, the slides were first placed in the Slide Cassette (from the Visium Slide kit) for the optimal permeabilization time. A permeabilization enzyme (from the Visium Reagent kit) was used for permeabilizing the tissue sections on the slide for incubating for the predetermined permeabilization time. The polyadenylated mRNA released from the overlying cells was captured by the primers on the spots. After washing by 0.0.1×SSC (saline sodium citrate buffer, MilliporeSigma), RT Master Mix (provided in Visium Reagent kit) containing reverse transcription reagents was added to the permeabilized tissue sections in the Thermocycler Adaptor. Incubation with the reagents produces spatially barcoded full-length cDNA from polyadenylated mRNA on the slide.

### Second strand synthesis and denaturation

After removing RT Master Mix (provided in Visium Reagent kit) from the wells, sections were incubated in 0.08 M KOH for 5 min and washed by Buffer EB (QIAGEN). Then, Second Strand Mix (provided in Visium Reagent kit) was added to the tissue sections on the slide to initiate second strand synthesis on the Thermocycler Adaptor. This is followed by denaturation and transfer of the cDNA from each capture area to a corresponding tube for amplification and library construction. The slides were washed by Buffer EB and incubated in 0.08 M KOH for 5 min. Then, samples from each well were transferred to a corresponding tube containing tris-HCl (1 M;pH 7.0) in eight-tube strip for amplification and library construction.

### cDNA amplification and quality control

Denaturation sample of 1xl was transferred to the quantitative polymerase chain reaction (qPCR) plate well containing the qPCR Mix [nuclease-free water + KAPA SYBR FAST qPCR Master Mix (KAPA Biosystems) + cDNA Primers (from Visium Reagent kit)]. The *C*q value for each sample was recorded after qPCR. For cDNA amplification, cDNA Amplification Mix (from Visium Reagent kit) was added to the remaining sample from denaturation. Then, the product was incubated in Thermocycler Adaptor for a cycle. For cDNA Cleanup-SPRIselect, 60 μl of SPRIselect reagent (Beckman Coulter) was added to each sample and incubated for 5 min at room temperature. The sample was repeatedly adsorbed by the magnet·High, washed with ethanol (MilliporeSigma) and Buffer EB and transferred to a new tube strip. Then, we ran 1 μl of sample on an Agilent Bioanalyzer High Sensitivity chip (Agilent, catalog no. 50674626) for cDNA quality control (QC) and quantification.

### Visium spatial gene expression library construction

Enzymatic fragmentation and size selection were used to optimize the cDNA amplicon size. Sample indexes and TruSeq Read 2 (read 2 primer sequence) were added via End Repair, A-tailing, Adaptor Ligation, and PCR. The final libraries contain the P5 and P7 primers used in Illumina amplification. Library construction was performed with Library Construction Kit (10x Genomics, catalog no. PN-1000190).

### Raw sequencing data processing

The Visium Spatial RNA-seq output and bright-field and fluorescence microscope images were analyzed by Space Ranger (version 1.1.0) to detect tissue, align reads, generate feature-spot matrices, perform clustering and gene expression analysis, and place spots in spatial context on the slide image. These pipelines combined Visium-specific algorithms with the widely used RNA-seq aligner STAR.

### ST data analysis

The Visium ST raw-seq were processed with the Space Ranger pipelinenomics). Then the data was scaled and normalized via the function scTransform, integrated via CCA, reduce dimensions through PCA and U-MAP, unsupervised clusters via FindNeighbors and FindClusters with resolution = 0.3 and identified different expression spots based on R package Seurat. Spatial co-localization and cellular niche used Seurat and mistyR (65).

### Statistical analysis

The R v4.2.2 was applied to conduct all statistical analyses in this study. The Wilcoxon test was implemented to compare the GSVA scores and immune infiltration between two groups. Spearman correlation analysis was used to investigate the relationship. The false discovery rate (FDR) was calculated by the Benjamini-Hochberg procedure (B-H). The log-rank test was utilized to assess the significance of observed differences in overall survival (OS) by survminer (66). Statistical significance was determined by *p <*0.05, unless explicitly specified otherwise.

## Data Availability

The datasets presented in this study can be found in online repositories. The names of the repository/repositories and accession number(s) can be found in the article/**Supplementary Material**.
