# Supplementary material for: Integrated analysis of single-cell, spatial and bulk RNA-sequencing identifies a cell-death signature for predicting the outcomes of head and neck cancer
Source: Front Immunol. 2024 Nov 7;15:1487966. doi: 10.3389/fimmu.2024.1487966 (PMC11578999; doi:10.3389/fimmu.2024.1487966)

**Supplementary figures and figure legends**


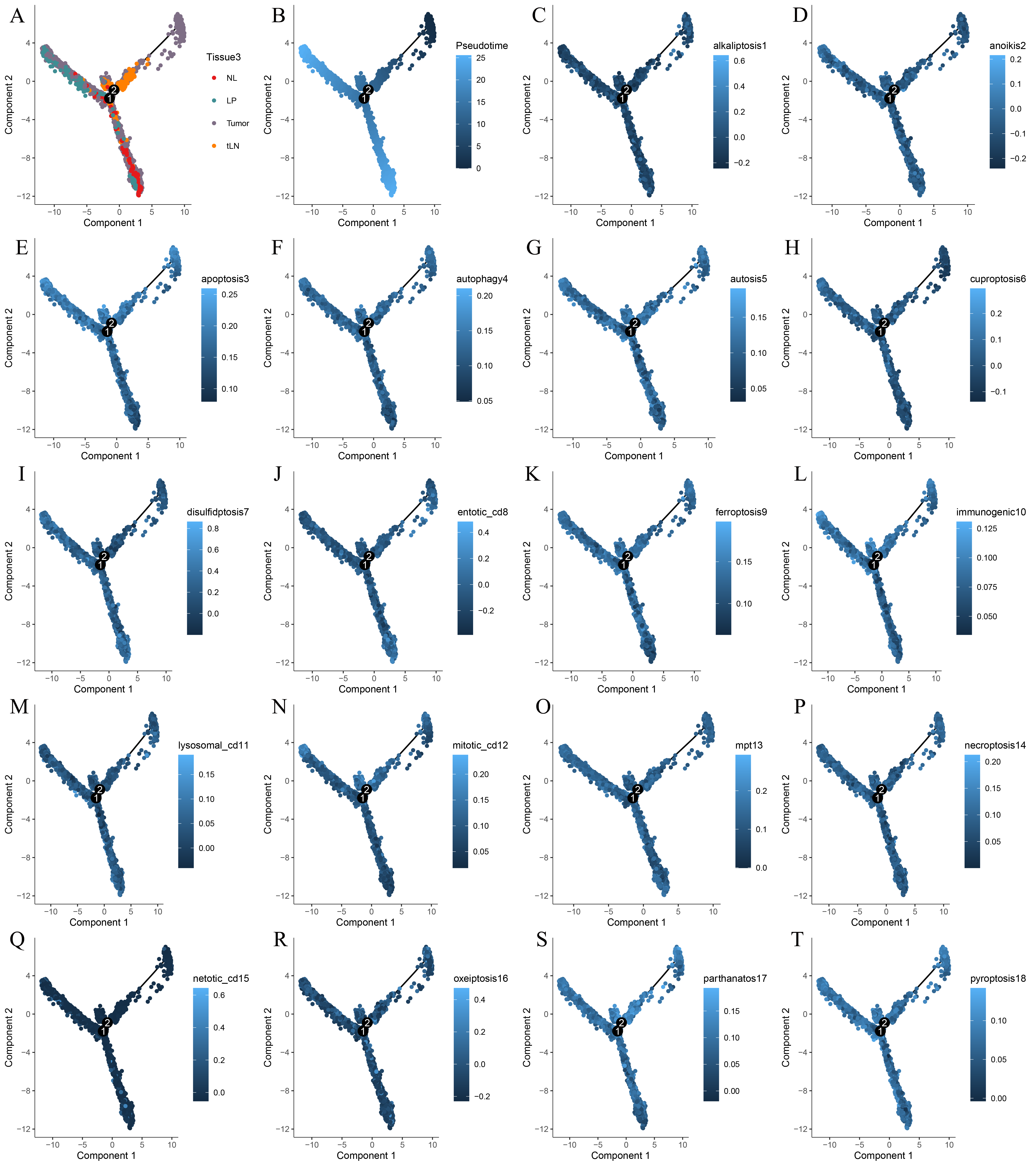


**Fig S1. Pseudotime analysis to evaluate the distribution of different cell death in epithelial/malignant cells.** The cell trajectory of epithelial/malignant cells generated by monocle2 is colored by each tissues (**A**), pseudotime score (**B**) and different cell death (**C-T**).


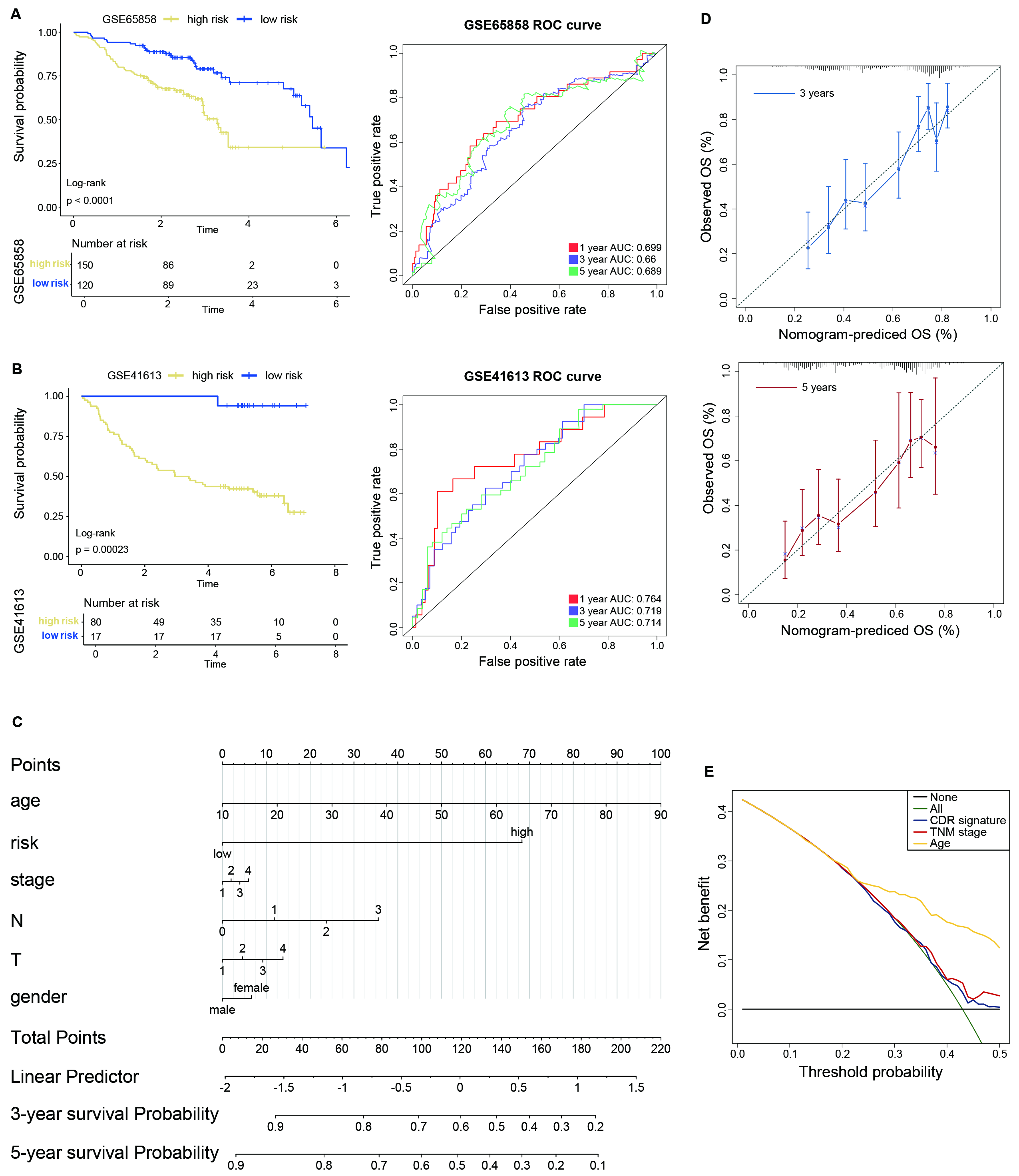


**sFig. 2** **Valitation of our consensus CDR signature**. (**A**) Kaplan-Meier curves of OS according to the RCD signature in GSE65858 and ROC curves for the RCD signature at 1,3 and 5 years. (**B**) Kaplan-Meier curves of OS according to the RCD signature in GSE41613 and ROC curves for the RCD signature at 1,3 and 5 years. (**C**) Construction of a nomogram. (**D**) Calibration curve of the nomogram for 3 and 5 years, respectively. (**E**) Decision curve of the nomogram.


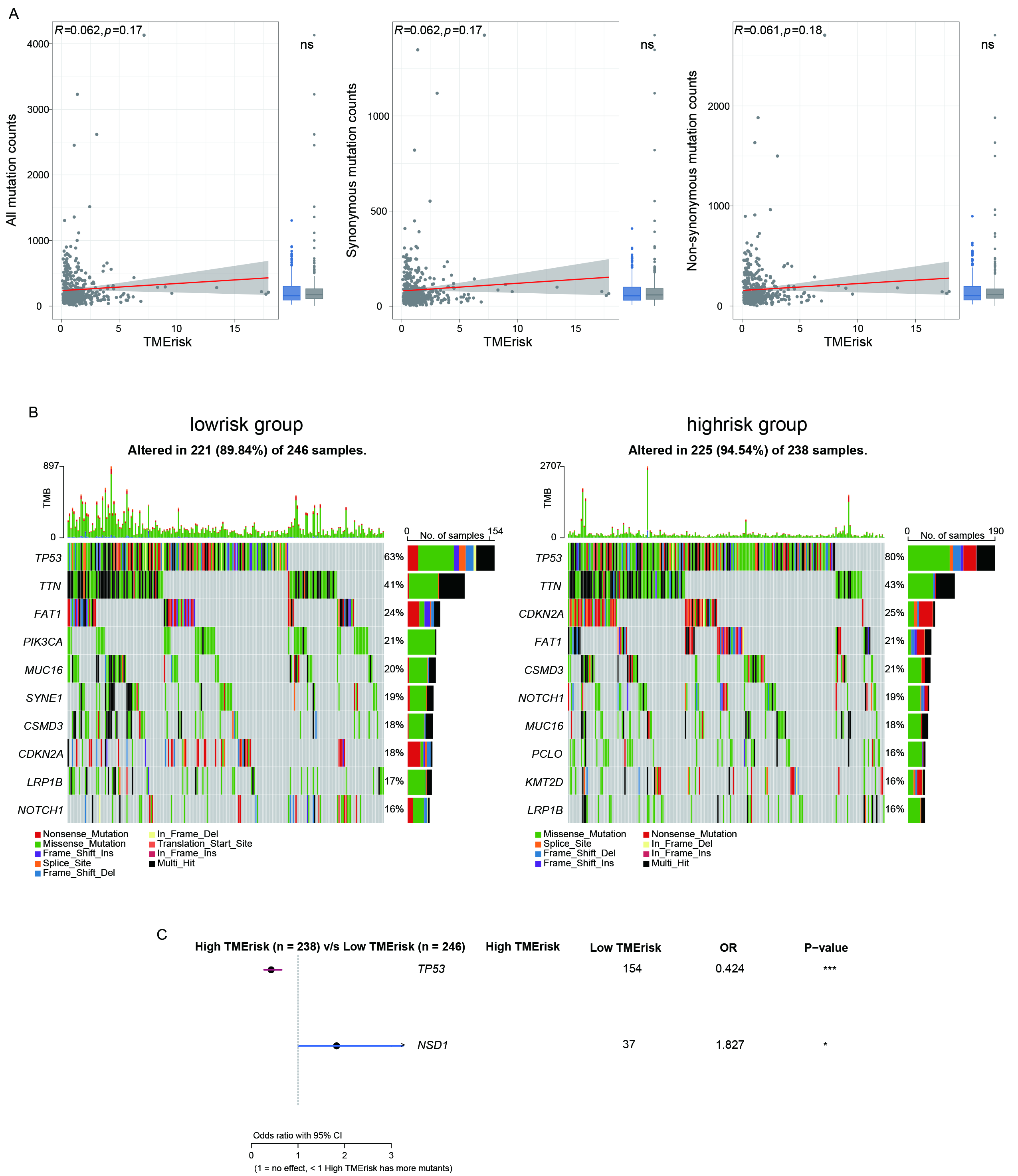


**sFig. 3** **The frequency of gene mutation in high- and low- risk score groups.** (**A**) patients were categorized into the high- and low- risk score groups, and the counts of all mutation counts (left), synonymous (middle), and non-synonymous (right) between these two groups was compared. NS, not significant different (*p* > 0.05). (B) Oncoprin showed the top ten genes mutation in the high- (left) and low- (right) risk score groups. (**C**) Forest plot showed the genes mutating differentially in patients of the low- and the high- risk score groups. **p*<0.05, ****p*<0.0001.

**sFig. 4** **The distribution and expression of these ten genes associated to our risk model at single cell level.** (**A**) UMAP projection of genes from our RCD signature. (**B**) Violin plots showing **expression levels of** genes from RCD signature **for each cell type among different tissues**.


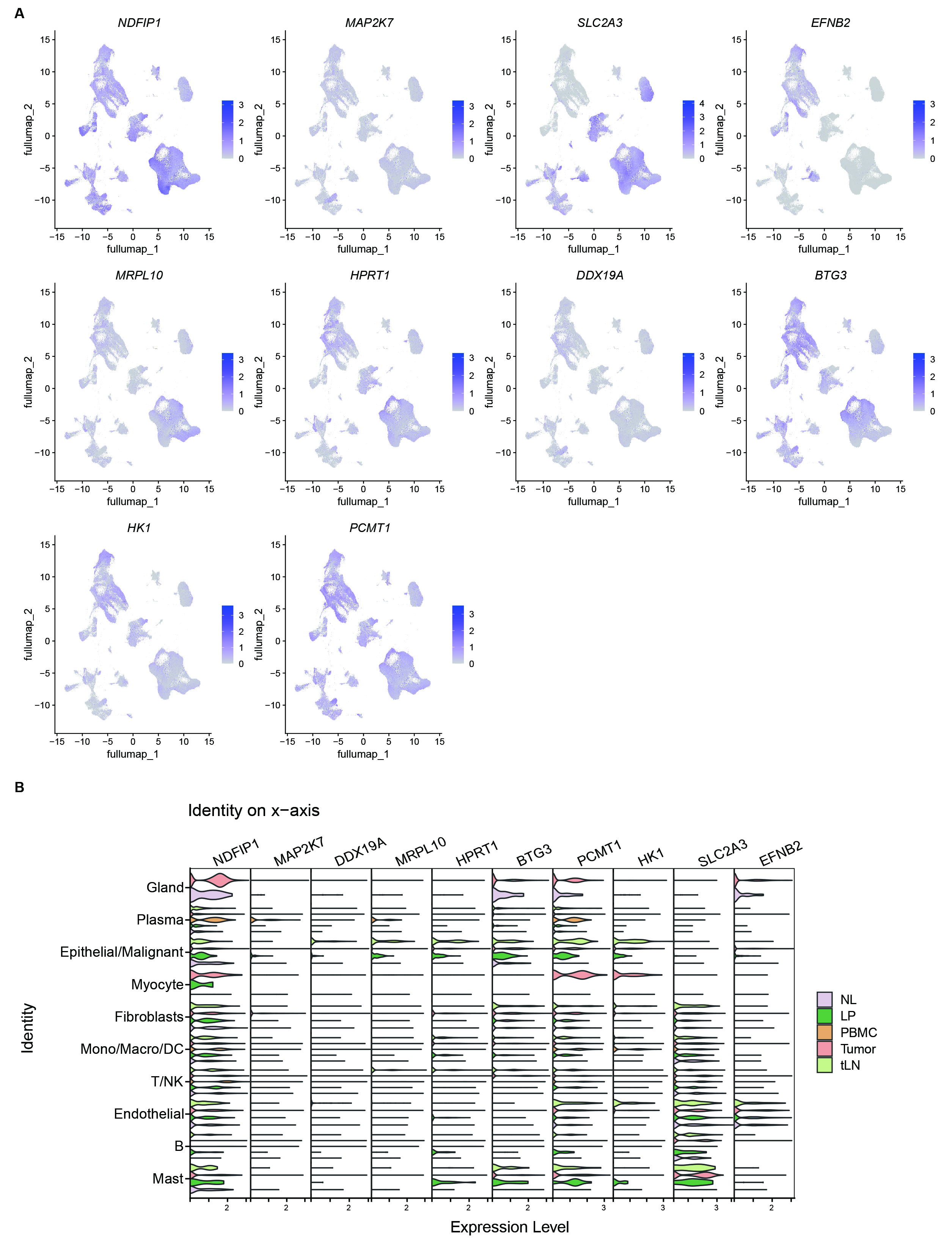

Supplement: Supplementary file 1 [file DataSheet1.docx]
